# Immediate dilation of a tight or stenotic cervix by intra-procedural administration of hyoscine butylbromide: A clinical trial

**DOI:** 10.18502/ijrm.v17i4.4550

**Published:** 2019-06-13

**Authors:** Shiva Hadadianpour, Shahrzad Tavana, Anahita Tavana, Masoumeh Fallahian

**Affiliations:** ^1^ Preventative Gynecology Research Center, Shahid Beheshti University of Medical Sciences, Tehran, Iran.; ^2^ University of Texas South West (UTSW), Dallas, Texas, USA.; ^3^ University of Texas Medical Branch (UTMB), Texas, USA.

**Keywords:** *Hyoscine butylbromide*, *Cervical dilatation*, * Cervical ripening*

## Abstract

**Background:**

Cervical dilation is indicated prior to performing various gynecological procedures. However, gynecologists are at times confronted with a stenotic or tight cervix, resistant to dilation. This can be problematic particularly when cervical ripening has not been attempted hours before the start of the procedure.

**Objective:**

The objective of this study is to investigate the efficacy of administration of hyoscine butylbromide for cervical dilation for immediate dilation of the tight or stenotic cervix.

**Materials and Methods:**

In this clinical trial study, a population of 40 women, aged 20-70 yr with stenotic cervix, evidenced by resistance to pass dilator #2 through their cervical canal were compared. Cervical patency was assessed 10 min following intra-cervical canal instillation of hyoscine butylbromide.

**Results:**

Cervical width of 57.5% of patients became wider, as evidenced by passage of the number 4 Hegar dilator through the cervical canal without resistance. Independent *T*-tests did not reveal any statistically significant difference between the two groups based on their age. Fisher Exact test revealed a statistically significant difference between the two groups based on the prior route of delivery, with a more statistically significant response in patients who had vaginal deliveries.

**Conclusion:**

Intra-cervical canal instillation of hyoscine butylbromide is effective in immediate dilation of the tight or stenotic cervix during intra-uterine procedures.

## 1. Introduction

Cervical dilation, a process naturally reserved for childbirth, is a process that is indicated for various gynecological procedures, including, but not limited to, endometrial biopsy, hysteroscopy, endometrial ablation, intrauterine device insertion, intrauterine insemination, etc. It is known that an unfavorable cervix, i.e., a non-primed and non-dilated cervix, poses a great challenge during these procedures. Prior to the initiation of gynecological procedures, various methods can be used to achieve ripening of the cervix. The two main methods of cervical ripening are chemical vs mechanical dilators. Mechanical dilators such as the traditional Hern, Hegar, Pratt, Hanks, and Denniston dilators are used to sequentially dilate the cervix (1). However, these dilators have been shown to be associated with numerous complications, such as uterine perforation, cervical laceration, infections, and intraperitoneal hemorrhage (2). Chemical dilators, such as prostaglandin E1 analogues, have been shown to facilitate cervical dilation and minimize cervical or uterine injuries during gynecological procedures such as intra-uterine device insertion (3). They have been formed based on the concept of the critical role the prostaglandins play in cervical ripening by increasing inflammatory mediators in the cervix and inducing cervical remodeling. Similarly, inhibitory impulses can lead to stenosis of the cervix, further posing a challenge during gynecological procedures (4). However, it is important to keep in mind that cervical priming with chemical agents is typically carried out 2–4 hr prior to the initiation of the procedure. Another chemical agent that has been shown to be effective in cervical dilation is hyoscine butylbromide. Hyoscine butylbromide, under the trade name of Buscopan, belongs to a para-sympatholytic group of drugs, and it is a semisynthetic derivative of Scopolamine. Due to its anti-cholinergic properties and high affinity for muscarinic receptors on smooth muscles, with a selectivity on cervico-uterine plexus, it can lead to dilation of a tight or stenotic cervix. Hyoscine butylbromide has a half-life of 4.5–5 hr (5). Its adverse effects following oral administration include skin reaction, tachycardia, vascular disorders (Buscopan injection), dry mouth, urinary retention, and gastric irritation, which are dose-dependent. However, these side effects could be mitigated by vaginal administration of hyoscine butylbromide, leading to a local response and a more selective effect on the cervix (6). Originally, the effect of hyoscine butylbromide was investigated for cervical dilation during the active phase of labor (7). It was shown that it can reduce the duration of the first stage of labor by overcoming cervical spasm, through its effect on blockage of transmission of neural impulses in the intraneural parasympathetic ganglia of abdominal organs (8). Our previous study demonstrated the efficacy of hyoscine butylbromide in cervical dilation in pre-menopausal women compared to control group when administered 8 hours and then 2 hours prior to the start of intrauterine gynecological procedures (6). However, the application of the mentioned pharmacologic agents for cervical ripening is time-consuming, making them unsuitable for use for immediate dilation of the cervix.

Our study aims to test the efficacy of intra-operative or procedural instillation of hyoscine butylbromide in the cervical canal for immediate dilation of the cervix during gynecologic procedures.

## 2. Materials and Methods

### Study patients

The study was conducted at the Department of Obstetrics and Gynecology at Sirjan Gharazi Hospital in Kerman, Iran from February 2018 to March 2018. Patients who were enrolled in this trial met the following criteria: women not pregnant at the time of the study, the age range of 20–70 yr, in general, good health, and stenotic cervix determined by obstetrics exam-scheduled for intrauterine procedures due to a gynecological disorder or for intra-uterine device insertion. Patients who had any of the following criteria were not enrolled in the study: prior hypersensitivity to hyoscine butylbromide, heart disease, thyroid disease, or hypertension, the temperature of 38ºC or higher, uterine pain, or any odorous vaginal secretions. Of note, no patient was on hormonal replacement therapy at the time of the study. In addition, no patient in the study had any history of cervical conization, loop electrical excision procedure, or cervical incompetency.

### Study design

The study included 40 women based on the mentioned criteria. Selection of the patients was based on the history and obstetrics exam that was carried out by the attending physician on the patient in lithotomy position immediately prior to the start of the procedure. Cervical patency was measured using dilator #2. Those patients in whom the dilator was not able to pass through their cervical canal, indicating a stenotic cervix, were included in this trial. Hyoscine Butyl Bromide (Vial 20 mg, Osve Company, Iran) under the trade name of Buscopan via instillation to the cervical ostium was administered to each patient. Each patient received contents of the 20 mg vial as a single dose. Cervical patency was assessed 10 min following the administration of hyoscine butylbromide, based on the diameter of the largest Hegar dilator that could be inserted into the cervix without any resistance.

### Ethical consideration

Prior to the enrollment in the trial, appropriate written informed consent thoroughly explaining the purpose of the study, advantages, and disadvantages of hyoscine butylbromide administration were obtained from each patient. The clinical trial was approved by the Ethics Committee of Shahid Beheshti University of Medical Science (IR.SBMU.RETECH.REC.1396.623) and it was registered within the Iranian registry of clinical trials.

### Statistical analysis

The data were analyzed using SPSS (Statistical Package for the Social Sciences, version 21.0 SPSS Inc, Chicago, Illinois, USA); Chi-Square test, Mann-Whitney U-test, and *T*-Independent test were used to compare the results with a statistically significant result threshold p < 0.05.

## 3. Results

The demographic characteristics of the study patients are listed in Table I. Among the 40 patients who participated in this study, 10 min following the administration of hyoscine butylbromide, cervical width of 23 out of 40 women (57.5%) became wider, as evidenced by passage of number 4 Hegar dilator through the cervical canal without resistance, 10 minutes after intra-cervical canal instillation of Hyoscine, became wider. The remaining 17 patients had no change in their cervical width in the first 10 minutes following the administration of hyoscine butylbromide.

The average age of patients who had dilation of the cervix was lower than the group with no cervical dilation - 37 yr vs. 43 yr. However, Independent *T*-test was performed on the two groups: 23 women who responded to hyoscine butylbromide administration and the 17 who did not have any change, based on their age. The results did not reveal any significant difference between the two groups based on age (Table II). Fisher Exact test revealed a significant difference between the response of women based on their prior route of delivery. Women who had a natural delivery in the past responded to the drug in the best way (75%) in comparison to the women who had only had cesarean delivery in their past history (60%), and the least response corresponded to the group of patients who were nulliparous (p = 0.013) (Table III).

Mann-Whitney U-test pointed out that the number of the previous pregnancy, any prior history of vaginal delivery, or history of C-section did not affect response to hyoscine butylbromide administration. This is despite the fact that previously we proved that prior route of delivery, not taking to account the number of deliveries, has a statistically significant influence on response to hyoscine butylbromide.

Following the application of the drug, the cervical width of premenopausal women changed in 19 out of 29 women but 4 out of 11 menopausal women showed this shift (p = 0.153) (Table IV).

Furthermore, Fisher Exact test did not reveal any statistically significant difference in cervical width between pre-menopausal and post-menopausal women (Table V).

**Figure 1:**
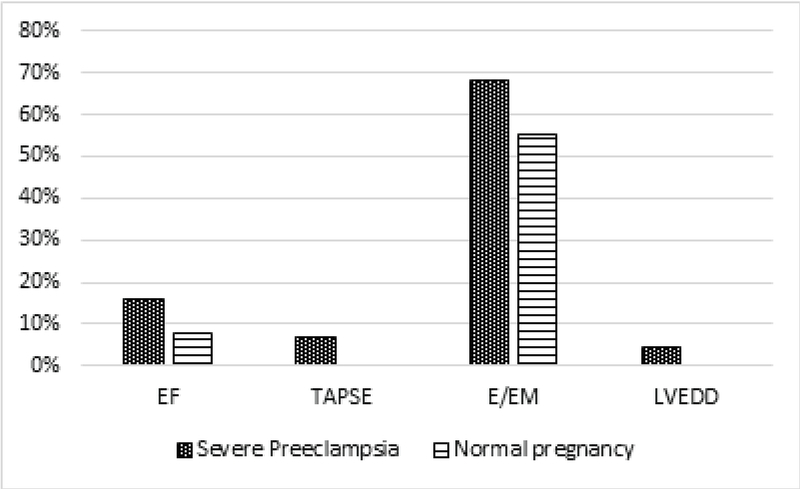
Study Design and Summary of Results based on age, history of vaginal delivery, and menopausal status.

Figure 1 summarized the study design and the results of the dilation of cervix based on age, menopausal status, and history of prior vaginal delivery.

**Table 1:** Independent *T*-test results on two groups with/without a change in cervical width after the administration of hyoscine butylbromide and average age of each group


**Hegar dilator #**	**Number**	**Average age**	**Std. deviation**	**Std. error mean**	**T-test**
2.0	17	43.706	17.6308	4.2761	
4.0	23	36.783	13.3313	2.7798	0.165

**Table 2:** Fisher exact test on cervical width after the administration of hyoscine butylbromide based on prior route of delivery


	**Hegar #2 Number of patients**	**Hegar #4 Number of patients**	**Total Number of patients**	**P-Value Fisher**
Nulliparous	8	1	9	
Vaginal delivery	3	9	12	
C-section	4	10	14	
Both vaginal delivery and C-section	2	3	5	0.013

**Table 3:** Mann-Whitney U-test on cervical width after the administration of hyoscine butylbromide based on child birth status


	**Hegar #2 Number of patients**	**Hegar #4 Number of patients**	**Total Number of patients**	**Mann-Whitney U-test p-value**
Number of Pregnancies				
0	8	1	9	
1	2	8	10	
2	2	7	9	
3	4	4	8	
4	1	1	2	
5	0	2	2	0.073
Number of vaginal deliveries				
0	12	11	23		
1	2	6	8		
2	1	1	2		
3	1	2	3		
4	1	1	2	
5	0	2	2	0.174
Number of C-sections				
0	11	10	21		
1	2	7	9		
2	3	5	8		
3	1	1	2		0.331

**Table 4:** Cervical width after the administration of hyoscine butylbromide based on menopausal state


	<**Cervical width based on Hegar dilator # following the administration of hyoscine butylbromide**		**Fisher exact test**
	2.0	4.0	
	Count	Count	
Pre-menopause	10	19	
Post-menopause	7	4	0.153

**Table 5:** Demographic characteristics of the study patients


	**Count**
lParity Status:	
Nulliparous	9
Vaginal Delivery	12
C-section	14
Both C-section and Vaginal Delivery	5
lMenopause Status:	
Pre-menopause	29
Post-menopause	11
Average Age of Participants	40.244
Total Number of Patients:	40

## 4. Discussion

In this clinical trial, intra-cervical canal instillation of hyoscine butylbromide is effective in immediate dilation of the tight or stenotic cervix during intra-uterine procedures, as evidenced by the dilation of the cervix in 57% of cases. We observed that the cervical dilation was not different in pre-menopausal vs post-menopausal women. Our results also indicated that prior route of delivery does affect cervical dilation, with more significant results in patients with prior history of vaginal delivery as opposed to Caesarian section; however, the number of deliveries did not significantly affect the response to hyoscine butylbromide administration. Previously, several studies had introduced various methods for dilation of the cervix for gynecological procedures. Examples include the use of mechanical dilation on a non-primed cervix utilizing the traditional Hern, Hegar, Pratt, Hanks, and Denniston dilators, the use of which, as mentioned before, is associated with higher rates of complications including, but not limited to, cervical tears, creation of false passages, and uterine perforation (2). In addition, alternative methods for cervical dilation were introduced in several studies using pharmacological methods, such as prostaglandin E1 analogue - misoprostol (3) - or hyoscine butylbromide (6), which involves a lower risk of complications compared to mechanical dilators, but requires administration several hours prior to the start of gynecological procedures in order to be effective. The study had indicated that the administration of hyoscine butylbromide hours prior to the procedure was effective in dilating the cervix in pre-menopausal, but not in post-menopausal women, and the difference was attributed to the likely difference in the cervical tissue collagen component and gonadal steroid deficiency (6). In the same study, our results had indicated that the administration of vitamin B6 as placebo did not lead to statistically significant dilation of the cervix when compared to hyoscine butylbromide administration (6). The importance of our current study is that it has investigated and introduced intra-cervical canal instillation of hyoscine butylbromide as a novel method for immediate dilation of a stenotic or tight cervix during the operation due to its anticholinergic effects on smooth muscles and its selectivity for the cervico-uterine plexus. A prior study had demonstrated efficacy of intramuscular administration of hyoscine butylbromide on reducing the duration of first stage of labor through hastening cervical effacement and dilation in primiparous women without labor augmentation, with no associated increased risk of complications (9). However, due to the presence of rich cervical blood supply, the use of hyoscine butylbromide through direct administration to the cervical ostium substantially increases its efficacy on dilating the cervix. Although the use of the drug through this route has a lot of benefits, the effect of hyoscine given in this way has rarely been studied.

Based on the results of this study, in more than half of the cases, there was no need for delaying the gynecological procedure hours after instillation of hyoscine butylbromide to observe its efficacy in dilating the cervix, as the effects were observed immediately after the administration during the procedure.

It is notable that because of the limited number of patients enrolled in this study, a large prospective study should be conducted in the future to further confirm the effectiveness of hyoscine butylbromide.

## 5. Conclusion

Intra-cervical canal instillation of hyoscine butylbromide is shown to have been effective in more than half of the study patients in immediate dilation of the tight and stenotic cervix during intra-uterine procedures.

##  Conflict of Interest

The authors declare no conflict of interest.
